# Therapeutic Approach Based on Nanotechnology with Chitosan-Coated Zein Nanoparticles Containing Quercetin Against Resistant *Klebsiella pneumoniae* Clinical Isolates

**DOI:** 10.3390/pharmaceutics17091227

**Published:** 2025-09-22

**Authors:** Azael Francisco Silva-Neto, Maria Anndressa Alves Agreles, Ana Alice Venancio Correia, Hanne Lazla Rafael de Queiroz Macêdo, Alane Rafaela de Carvalho Amaral, Alexsandra Maria Lima Scavuzzi, João Victor de Oliveira Alves, Ana Catarina Souza Lopes, Márcia Vanusa da Silva, Maria Tereza dos Santos Correia, Isabella Macário Ferro Cavalcanti, Luís André de Almeida Campos

**Affiliations:** 1 Keizo Asami Institute (iLIKA), Federal University of Pernambuco (UFPE), Recife 50670-901, PE, Brazil; azael.silvaneto@ufpe.br (A.F.S.-N.); anndressa.agreles@ufpe.br (M.A.A.A.); alice.venancio@ufpe.br (A.A.V.C.); hanne.queiroz@ufpe.br (H.L.R.d.Q.M.); 2 Department of Pharmaceutical Sciences, Federal University of Pernambuco (UFPE), Recife 50670-901, PE, Brazil; alane.carvalho@ufpe.br; 3 Laboratory of Microbiology, Department of Tropical Medicine, Federal University of Pernambuco, Recife 50670-901, PE, Brazil; alexsandramariah@gmail.com (A.M.L.S.); ppg.medtrop@ufpe.br (A.C.S.L.); 4 Department of Biochemistry, Federal University of Pernambuco, Recife 50670-901, PE, Brazil; joao.oliveiraalves@ufpe.br (J.V.d.O.A.); marciavanusa@yahoo.com.br (M.V.d.S.); coordenacao.ppgcb@ufpe.br (M.T.d.S.C.); 5 Laboratory of Microbiology & Immunology, Academic Center of Vitoria, Federal University of Pernambuco, Vitória de Santo Antão 55608-680, PE, Brazil; 6 Laboratory of Microbiology and Parasitology, Campus Ouricuri, University of Pernambuco, Ouricuri 56200-000, PE, Brazil

**Keywords:** antibacterial therapy, biofilms, bacterial resistance, nanostructures

## Abstract

**Background/Objectives:** The study developed, characterized, and evaluated the toxicity, antibacterial and antibiofilm activity of quercetin encapsulated in chitosan-coated zein nanoparticles (QUER-ZNP-CH). **Methods:** QUER-ZNP-CH were prepared by the nanoprecipitation method and characterized by physicochemical analyses, stability (12 months), and release kinetics. Toxicity was evaluated through hemocompatibility and a *Tenebrio molitor* larval model. Antibacterial activity (MIC/MBC, CLSI) and antibiofilm potential (crystal violet assay) were tested against resistant *Klebsiella pneumoniae* strains. **Results:** The nanoparticles were prepared, and physicochemical analyses revealed chemical interactions, efficient encapsulation of the drug, and thermal stability. The formulations remained stable over 12 months, and the release kinetics demonstrated controlled release for 72 h. No hemotoxic profile was observed and there was 95% survival of *Tenebrio molitor* larvae after treatment with QUER-ZNP-CH. The minimum inhibitory concentration (MIC) and minimum bactericidal concentration (MBC) of QUER-ZNP-CH revealed enhanced antibacterial activity of QUER, as indicated by a 32 to 64-fold reduction in the MIC and MBC values. The biofilm inhibition potential of QUER-ZNP-CH showed 60–100% inhibition and 25–95% eradication in concentrations from 0.12 to 62.5 μg/mL. **Conclusions:** Thus, this nanotechnology-based formulation suggests potential for the treatment of bacterial infections caused by multidrug-resistant *K. pneumoniae* strains.

## 1. Introduction

*Klebsiella pneumoniae* is an opportunistic Gram-negative bacillus of clinical relevance, associated with respiratory tract, urinary tract, wound, intra-abdominal and soft tissue infections, sepsis, meningitis, and healthcare-associated infections (HAIs). These infections are particularly prevalent among patients with comorbidities, neonates, the elderly, immunocompromised or immunosuppressed individuals [1,2]. Moreover, a notable increase in antimicrobial resistance has been observed in *K. pneumoniae* strains, primarily through the expression of efflux pumps and the production of extended-spectrum β-lactamases (ESBLs) and carbapenemases (KPC), as well as other mechanisms such as porin loss and target modification. The various resistance mechanisms exhibited by this species against commonly used antimicrobials limits therapeutic options and represents a significant global public health concern [2,3,4].

In addition to antimicrobial resistance, *K. pneumoniae* has the ability to form biofilms, in which bacterial cells are embedded in a self-assembled matrix composed of polysaccharides, proteins, and DNA [5]. This extracellular matrix impedes drug penetration and action while facilitating pathogen adherence to catheters and the internal surfaces of medical devices, posing an additional therapeutic challenge in managing these infections [5,6].

In this context, the use of plant-derived compounds, particularly flavonoids, has emerged as a promising strategy to overcome bacterial resistance, inhibit biofilm formation, and promote biofilm eradication [7,8]. Among natural flavonoids, quercetin (QUER) stands out as a flavonol with well-established antioxidant, anti-inflammatory, antimicrobial, and antibiofilm properties. QUER can inhibit bacterial adhesion, disrupt biofilm architecture, and modulate virulence factors, making it a valuable candidate for therapeutic development. Additionally, recent meta-analyses have confirmed the reno-protective effects of quercetin in preclinical models of acute kidney injury, supporting its pharmacological relevance for systemic infections and inflammation-associated tissue damage [9]. However, its exhibits poor water solubility and low oral bioavailability, which restricting its direct application [10,11,12].

Therefore, the encapsulation of QUER into nanocarrier systems has been proposed as a promising technological approach to enable its application in the treatment of infections caused by multidrug-resistant bacteria [13,14]. Recent advances in allergen-based nanobiotechnology have further demonstrated that molecular encapsulation and immune-modulation strategies can enhance therapeutic potential beyond infectious diseases [15], underscoring the versatility of nanocarriers in modulating biological responses and highlighting their broader applicability in nano-based therapeutics.

Among the various nanocarriers described, the encapsulation of hydrophobic bioactive compounds in zein-based nanostructures has garnered growing attention within the scientific community. Zein, a corn-derived protein, possesses favorable physicochemical properties and chemical composition for the formation of nanospheres, enabling the entrapment of hydrophobic molecules within its polymeric matrix. This contributes to enhanced stability and controlled release upon oral, nasal, or intravenous administration [16,17,18].

Furthermore, the surface functionalization of zein nanoparticles with chitosan confers additional protection to the encapsulated molecule, promoting controlled release within the organism and enhancing the therapeutic efficacy of the active compounds [17,19,20]. Moreover, chitosan-based nanocarriers have been reported to provide dual functions of remineralization and antibacterial activity in dental applications [21], underscoring their versatility as biomedical nanoplatforms capable of combining structural and therapeutic functionalities.

In this study, we aimed to develop and characterize chitosan-functionalized zein nanospheres loaded with QUER and to evaluate their in vitro hemotoxicity, toxicity in a *Tenebrio molitor* larval model, as well as their antibacterial and antibiofilm activities both in vitro and in vivo in a *Tenebrio molitor*. This approach is innovative as it is the first study to evaluate quercetin-loaded zein/chitosan NPs against resistant *K. pneumoniae* clinical isolates and provides a robust framework for exploring novel strategies to combat multidrug-resistant *K. pneumoniae* infections.

## 2. Materials and Methods

### 2.1. Materials

Culture media, QUER, and other reagents were obtained from Himedia^®^ (Mumbai, India) and Sigma-Aldrich^®^ (St. Louis, MO, USA). All solvents were supplied by Merck^®^ (Darmstadt, Germany). Five clinically resistant *Klebsiella pneumoniae* isolates originating from colonization (rectal swab), urinary tract infections, or respiratory tract infections were collected from three public hospitals in Recife, Pernambuco, Brazil [22], and were kindly provided by Prof. Dr. Ana Catarina Lopes from the Department of Tropical Medicine at the Federal University of Pernambuco (UFPE). In addition, two ATCC strains were used: *K. pneumoniae* ATCC 13883, employed as the standard strain for microbiological studies, and ATCC 700603, used as a classical model of antimicrobial resistance. Both strains were obtained from the American Type Culture Collection (ATCC) (Manassas, VA, USA) and preserved at −80 °C in BHI broth supplemented with 20% glycerol.

### 2.2. Preparation of Chitosan-Coated Zein Nanoparticles Containing Quercetin (QUER-ZNP-CH)

Chitosan-coated zein nanospheres were prepared by nanoprecipitation, following a method adapted from Campos et al. [17]. Initially, zein (200 mg) and lysine (20 mg) were dissolved in 70% ethanol under magnetic stirring at 600 rpm for 30 min. Lysine was included to promote a slight alkalinization of the medium, facilitating zein solubilization and stabilizing nanoparticle formation. Simultaneously, QUER (12.5 mg) was solubilized in absolute ethanol and subsequently added to the zein/lysine solution, followed by an additional 30 min of stirring. Ultrapure water was then slowly added dropwise to the organic phase, inducing zein self-assembly and nanoparticle formation. The residual ethanol was removed by rotary evaporation, yielding a final suspension of approximately 25 mL. For coating, chitosan (powder; MW 50,000–190,000 Da; viscosity 20–300 cP in 1 wt.% acetic acid at 25 °C, Brookfield; degree of deacetylation ~100%; (Sigma-Aldrich, St. Louis, MO, USA) was used to prepare a 0.5% (*w*/*v*) aqueous solution, which was then added to the zein nanoparticle dispersion in a 5:1 (*v*/*v*) zein-to-chitosan ratio, under magnetic stirring for 1 h, resulting in chitosan-coated zein nanoparticles loaded with QUER (QUER-ZNP-CH). After preparation, the QUER-ZNP-CH were lyophilized using 5% mannitol as a cryoprotectant.

### 2.3. Formulation Characterization

#### 2.3.1. Particle Size, PDI, Zeta Potential, and pH

The physicochemical properties of the QUER-ZNP-CH were evaluated by photon correlation spectroscopy (dynamic light scattering, DLS) using a Zetasizer Nano-ZS90 (Malvern Instruments, Worcestershire, UK) at 25 °C in triplicate (*n* = 3 independent preparations). For hydrodynamic diameter analysis, 50 µL aliquots of the nanoparticle dispersion were diluted in 950 µL of purified water and measured at 25 °C with a fixed detection angle of 90°. Results were expressed as the mean particle size in nanometers (nm), accompanied by the polydispersity index (PDI). Zeta potential was determined after dilution of 50 µL of the formulation in 950 µL of ultrapure water, and values were expressed in millivolts (mV), indicating the surface charge of the nanoparticles. pH measurements were performed at room temperature using a glass electrode coupled to a digital pH meter (mPA-210P, MS Tecnopon, São Paulo, Brazil), as described by Campos et al. [17]. All analyses were conducted in triplicate, and the results are presented as the mean of three independent measurements ± standard deviation (SD).

#### 2.3.2. Encapsulation Efficiency (%EE)

To determine the %EE of QUER, an analytical calibration curve was constructed using standard solutions in ethanol at concentrations ranging from 2 to 18 μg/mL. The %EE was quantified using a combined ultrafiltration and ultracentrifugation method, employing centrifugal filter units (Amicon® Ultra, Millipore, Billerica, MA, USA). Aliquots (1000 μL) of the QUER-ZNP-CH formulation were transferred into the filters and centrifuged at 8000× *g* for 1 h. Subsequently, 60 μL of the filtrate was diluted in ethanol, filtered, and analyzed by high-performance liquid chromatography with UV detection (HPLC-UV) at 343 nm. Chromatographic analysis was performed using a C18 column (250 mm × 4.6 mm, 5 μm, XBridge Waters®, Milford, MA, USA). as the stationary phase. The mobile phase consisted of a 0.3% trichloroacetic acid aqueous solution (50:50, *v*/*v*) and acetonitrile (70:30, *v*/*v*), as previously described by Abdelkawy, Balyshev, and Elbarbry [23]. Measurements were performed in triplicate on separate days. The concentration of non-encapsulated QUER was determined, and the encapsulation efficiency was calculated using the following equation:

$$\%EE=\;\frac{Total\;of\;QUER\;-\;filtered\;from\;QUER}{Total\;of\;QUER}\;\times \;100$$

#### 2.3.3. Fourier-Transform Infrared Spectroscopy (FTIR)

Fourier-transform infrared (FTIR) spectra in the mid-infrared region were acquired using an IRXross spectrometer (Shimadzu, Tokyo, Japan) equipped with an attenuated total reflectance (ATR) accessory. Each analysis was performed with an average of 45 scans per sample, over the spectral range of 4000 to 600 cm^−1^. Atmospheric CO_2_ bands were suppressed, and the resulting spectra were subsequently normalized for comparative purposes.

#### 2.3.4. Thermal Analyses

Differential scanning calorimetry (DSC) and thermogravimetric analysis (TGA) were performed using DSC-60 Plus and DTG-60H instruments (Shimadzu^®^, Kyoto, Japan) under a nitrogen atmosphere (50 mL·min^−1^). Approximately 5 mg of each sample were weighed into aluminum crucibles for DSC or alumina crucibles for TGA. DSC analyses were carried out from 30 to 300 °C at a heating rate of 10 °C·min^−1^, while TGA analyses were conducted from 30 to 800 °C at 20 °C·min^−1^. Empty crucibles were used as reference, and system calibration was performed using indium as a standard.

#### 2.3.5. Nanoparticle Morphology

The morphology of the nanoparticles was analyzed by scanning electron microscopy (SEM). The QUER-ZNP-CH were diluted in ultrapure water at a 1:10 (*v*/*v*) ratio, deposited onto aluminum stubs, and dried in a desiccator at room temperature for 24 h. After drying, the samples were sputter-coated using a FINE COAT ION SPUTTER JFC-1100 coater (JEOL Ltd., Tokyo, Japan) and subsequently examined using a ZEISS EVO-LS15 scanning electron microscope (Carl Zeiss AG, Oberkochen, Germany) [17].

#### 2.3.6. Long-Term Physicochemical Stability of QUER-ZNP-CH

The nanoparticles (QUER-ZNP-CH) were stored in lyophilized form at 4 °C in the absence of light and humidity for 12 months and evaluated monthly to determine mean particle diameter, polydispersity index (PDI), zeta potential, and drug content.

#### 2.3.7. In Vitro Release Kinetics of QUER-ZNP-CH

The in vitro release kinetics profile of QUER was evaluated using the dialysis bag technique with cellulose membrane bags (molecular weight cut-off: 15–20 kDa; Sartorius, Göttingen, Germany) in phosphate-buffered saline (PBS, pH 7.4). The release assay was conducted under sink conditions, where the dissolution medium volume was ten times greater than the saturation volume. An aliquot of 3 mL of the QUER-ZNP-CH suspension was placed inside the dialysis membrane and immersed in 30 mL of PBS buffer (pH 7.4) under constant agitation (70 rpm) at 37 °C for 72 h. Aliquots of 3 mL were collected at predefined time intervals: initially at 15, 30, and 45 min during the first hour, followed by 1, 2, 3, 4, 5, 6, 7, 8, 9, 10, 11, 12, 16, 24, 30, 36, 40, 42, 48, 56, 64, and 72 h. After each sampling, the same volume (3 mL) of fresh PBS (pH 7.4) was added to maintain sink conditions. QUER content was quantified by UV detection via HPLC, as previously described [17].

### 2.4. In Vitro Antibacterial Activity

#### 2.4.1. Evaluation of Antibacterial Activity

Antibacterial activity was assessed by determining the minimum inhibitory concentration (MIC) using the broth microdilution method, in accordance with the guidelines of the Clinical and Laboratory Standards Institute [24]. Initially, 95 µL of Mueller-Hinton broth (MHB) was dispensed into U-bottom 96-well microplates. Free QUER was prepared in a DMSO 2% solution, and both QUER and QUER-ZNP-CH were then added to the first column of the microdilution plates. Serial two-fold dilutions were then performed across the plates, yielding concentrations ranging from 250 to 0.48 µg/mL.

Bacterial suspensions of *K. pneumoniae* ATCC 13883, *K. pneumoniae* ATCC 700603, and clinical isolates K25-A2, K26-A2, K29-A2, K31-A2, and K32-A2 were adjusted to a turbidity equivalent to 0.5 on the McFarland scale and inoculated into the wells containing the test samples and MHB. The plates were incubated at 35 ± 2 °C for 24 h. MIC values were determined as the lowest concentration for which there is no visible bacterial growth in the medium. Each plate included appropriate controls to ensure the reliability and interpretability of the results. One column served as the positive control, containing bacterial suspension without the test compounds to confirm normal bacterial growth. Another column served as the negative control, containing only MHB without bacteria or test compounds to confirm the sterility of the medium.

For determination of the minimum bactericidal concentration (MBC), aliquots from wells showing no visible growth were plated onto Mueller-Hinton agar (MHA) and incubated under the same conditions. The MBC was defined as the lowest concentration capable of completely eliminating bacterial growth [24]. All experiments were carried out in triplicates, independently, on separate days.

#### 2.4.2. In Vivo Antibacterial Assay with *Tenebrio molitor*

For the experimental design, healthy *Tenebrio molitor* larvae (last larval instar, ~100 mg) were obtained from a commercial supplier (Insetos Brasil—Recife, PE, Brazil) and maintained in plastic trays (60 × 40 × 12 cm) under a 12 h light and 12 h dark photoperiod at 26 ± 1 °C. The insects were fed ad libitum with wheat bran (12% protein, 2% lipids, 75% carbohydrates, 11% minerals/sugars), along with pieces of sugarcane (*Saccharum officinarum*) and chayote (*Sechium edule*), as described by Czarniewska et al. [25].

For the infection model, larvae were randomly divided into groups of 10 individuals. Each larva was injected with 10 μL of a suspension containing *K. pneumoniae* ATCC 13883 or K32-A2 (1 × 10^7^ CFU/mL) into the last left proleg using a sterile microsyringe. The strain selection was based on the best in vitro response observed, allowing for the evaluation of compound efficacy in a standardized and clinically relevant model, thereby enhancing the robustness of the results.

After 2 h of incubation at 37 °C, infected groups were treated with 10 μL of either QUER or QUER-ZNP-CH at the concentration corresponding to the previously determined in vitro MIC. Control groups consisted of: (i) infected larvae treated with NaCl 0.9% and (ii) uninfected larvae treated with the formulation (Negative infection control) [25]. Larval viability was monitored daily for eight days, with death defined as the absence of response to tactile stimulation. In addition, clinical signs such as cuticular darkening, lethargy, and paralysis were recorded [25]. All experiments were performed in triplicate, independently, on separate days.

### 2.5. Antibiofilm Activity

#### 2.5.1. Evaluation of Biofilm Formation Inhibition

Biofilm production by *K. pneumoniae* strains ATCC 13883, K26-A2, K29-A2, and K31-A2 was determined in a previous study conducted by Campos et al. [17]. Tryptic soy broth supplemented with 1% glucose (TSB + glucose) was distributed in flat-bottom microdilution plates. The QUER and QUER-ZNP-CH were added at subinhibitory concentrations (MIC/8, MIC/16, and MIC/32), at which no bacterial growth inhibition was observed. Bacterial suspensions (10^5^ CFU/mL) were then inoculated. Plates were incubated at 35 ± 2 °C for 24 h. After incubation, TSB was aspirated, and the wells were washed with phosphate buffer at a pH 7.4. The well plates were dried, and the attached bacteria were fixed with 99% methanol. After fixation, the methanol was removed from the wells, and the plates were dried again. Subsequently, the bacteria adhered to the plates were stained with 1% crystal violet, and the excess crystal violet was removed. The results were then analyzed by spectrophotometry at a wavelength of 570 nm [26]. Controls included: TSB without inoculum (negative control) and TSB with inoculum but no treatment (positive control). Results were expressed as a percentage of inhibition, as described by Correia et al. [27]. All experiments were performed in triplicate, independently, on separate days.

#### 2.5.2. Evaluation of Biofilm Eradication

The antibiofilm activity on established biofilms was evaluated using the same clinical strains. Bacterial inocula (0.5 McFarland standard) were prepared in TSB + glucose and distributed into microdilution plates, followed by incubation at 35 ± 2 °C for 24 h to allow biofilm formation. After this period, the medium was carefully removed and replaced with fresh medium containing the formulations QUER and QUER-ZNP-CH at supra-inhibitory concentrations (8× MIC, 4× MIC, 2× MIC and MIC), considering the therapeutic range without compromising application viability due to toxicity. The plates were re-incubated for another 24 h at 35 ± 2 °C. Sterility controls (TSB without inoculum) and growth controls (TSB with inoculum) were maintained. The residual biomass was quantified using crystal violet staining described above, and the results were expressed as a percentage of eradication, as described by Correia et al. [27]. All experiments were performed in triplicate, independently, on separate days.

#### 2.5.3. Scanning Electron Microscopy of Biofilm

The evaluation of biofilm eradication by Scanning Electron Microscopy (SEM) was carried out with adaptations according to Correia et al. [27]. Initially, a suspension of clinical isolate K26-A2 was adjusted to 0.5 on the McFarland scale. The samples were prepared using 14G catheters, which were placed in the wells of a flat-bottom 24-well plate and incubated for 24 h at 35 ± 2 °C to allow biofilm formation. After incubation, the culture medium was removed and replaced with fresh medium. Subsequently, QUER or QUER-ZNP were added to the microplates at a concentration of 8× MIC and incubated for another 24 h at 35 ± 2 °C.

After incubation, the samples were washed three times with PBS buffer and fixed in a solution containing 2.5% glutaraldehyde and 4% paraformaldehyde in 0.1 M cacodylate buffer at pH 7.2 for 12 h. Then, 1% osmium tetroxide (OsO_4_) was added for post-fixation, followed by a dehydration process using an ascending series of ethanol concentrations. After dehydration, the samples were subjected to critical point drying using liquid CO_2_ and then sputter-coated with gold. Bacteria not exposed to any treatment were used as a positive control for biofilm formation. The prepared samples were analyzed using a scanning electron microscope (SEM), model Jeol JSM-5600 (JEOL Ltd., Tokyo, Japan), operating at 15 kV.

### 2.6. In Vitro and In Vivo Toxicity

#### 2.6.1. Hemolytic Activity

Hemolytic assays of QUER, ZNP-CH and QUER-ZNP-CH were carried out according to Florenço et al. [28]. Initially, blood samples were centrifuged (1.400× *g* at 25 °C for 10 min). Then, the plasma was discarded, and the blood cells were diluted in saline to obtain a concentration of 2%. Next, the blood cells were exposed to QUER and QUER-ZNP-CH at concentrations ranging from 0.97 to 250 µg/mL. The samples were then incubated at 35 ± 2 °C for 1 h and centrifuged again (5.600× *g* at 25 °C for 1 h). Afterwards, the supernatants were transferred to microdilution plates and analyzed by spectrophotometry at 540 nm (Multiskan Microplate Photometer FC, Thermo Scientific, Madrid, Spain). The results were expressed as a percentage of hemolysis, considering 100% hemolysis as the red blood cells exposed to 0.2% Triton X-100 (Sigma-Aldrich, St. Louis, MO, USA). The negative control corresponded to the blood cells incubated with PBS (pH 7.4). The institutional ethics committee approved the study, as indicated by approval number (CAAE: 46976315.9.0000.5208). All experiments were performed in triplicate, independently, on separate days.

#### 2.6.2. Toxicity in the *Tenebrio molitor* Model

The experimental design was carried out as previously described [25]. For the toxicity test, larvae were randomly distributed into 10 groups of 10 individuals each. Each larva received an injection of 10 μL of QUER or QUER-ZNP-CH into the last pair of prolegs on the left side using a sterile microsyringe, at concentrations ranging from 0.97 to 250 µg/mL, corresponding to the concentrations used for antibacterial activity. Control groups consisted of: (i) larvae treated with NaCl 0.9%, (ii) larvae treated with non-encapsulated QUER, and (iii) larvae treated with QUER-ZNP-CH. Larval viability was monitored daily for eight days, with death defined as the absence of response to tactile stimulus. All experiments were performed in triplicate, independently, on separate days.

### 2.7. Statistical Analysis

The tests used to compare the means of multiple groups were performed using one-way or two-way analysis of variance (ANOVA), followed when appropriate by Tukey’s multiple comparison procedure in GraphPad Prism 5.0 software (GraphPad, San Diego, CA, USA). The statistical data were considered significant with *p* < 0.05.

## 3. Results

### 3.1. Nanoparticulate Formulation Characterization

#### 3.1.1. Physicochemical and Spectroscopic Characterization

The nanoparticles were characterized regarding average diameter (Ø), polydispersity index (PDI), zeta potential (ζ), pH, and encapsulation efficiency (%EE), as shown in Table 1.

**Figure 1:**
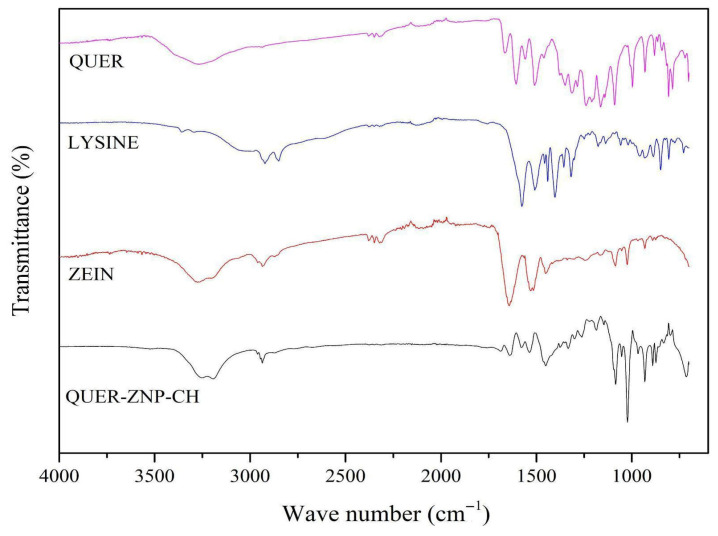
FTIR spectra of QUER, lysine, zein, and QUER-ZNP-CH.

The FTIR spectra (Figure 1) of the individual components (QUER, lysine, and zein) and the final formulation (QUER-ZNP-CH) showed characteristic bands for each substance. QUER exhibited peaks at approximately 3400 cm^−1^ (O–H), 1660 cm^−1^ (C=O), and 1600–1450 cm^−1^ (aromatic C=C). Zein displayed typical amide bands at around 1650 cm^−1^ and 1540 cm^−1^. In QUER-ZNP-CH, a reduction in intensity and shifts in QUER bands were observed, suggesting its encapsulation and interaction with the zein matrix and chitosan coating.

#### 3.1.2. Thermal Analyses

**Figure 2:**
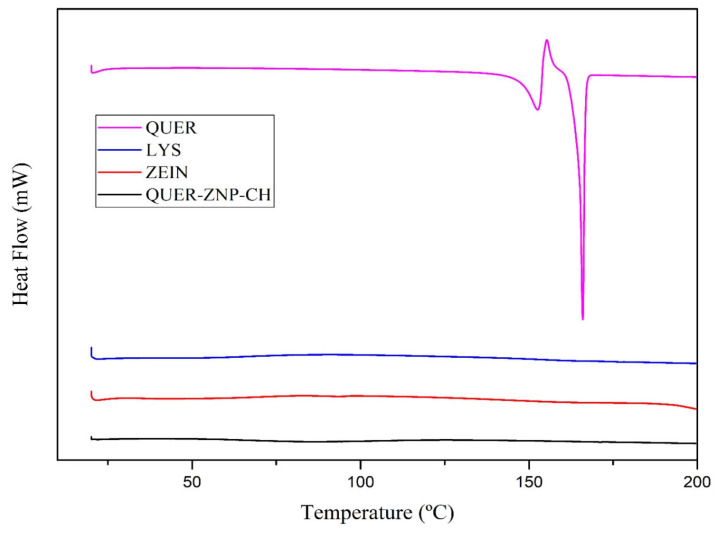
DSC curves of QUER, lysine, zein, and the QUER-ZNP-CH formulation.

**Figure 3:**
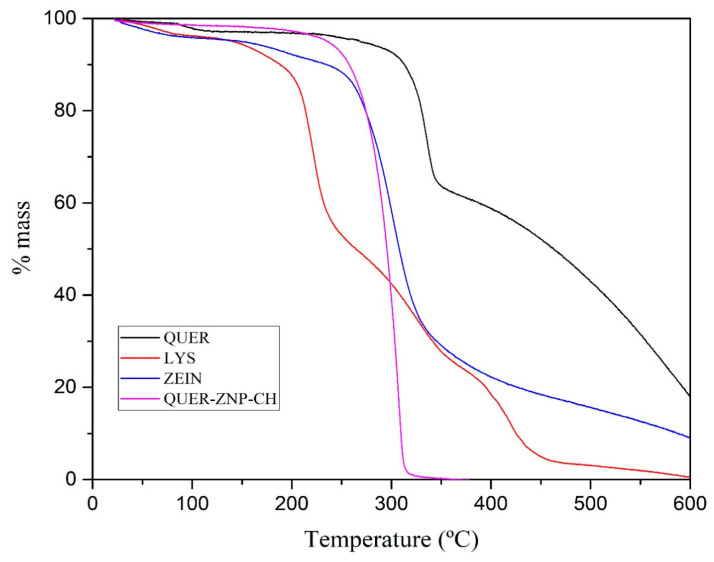
TG curves of QUER, lysine, zein, and the QUER-ZNP-CH formulation.

DSC thermal analyses (Figure 2) showed that QUER exhibited an endothermic peak between 170 and 180 °C, corresponding to its melting point, confirming its crystalline nature [29,30]. In contrast, the QUER-ZNP-CH formulation did not show this thermal event, indicating the absence of the drug’s crystalline form. The individual curves of lysine and zein also did not present defined thermal transitions in this range, showing only slight baseline variations. Thermogravimetric analysis (Figure 3) revealed that QUER exhibited high thermal stability, with degradation starting at around 320 °C. Zein began degrading around 270 °C, while lysine showed thermal instability, with mass loss starting at 200 °C. The QUER-ZNP-CH formulation began degrading at approximately 270–280 °C, with a sharp mass loss between 300 and 330 °C, demonstrating a unified and abrupt profile distinct from the isolated components [31,32].

#### 3.1.3. Nanoparticle Morphology

**Figure 4:**
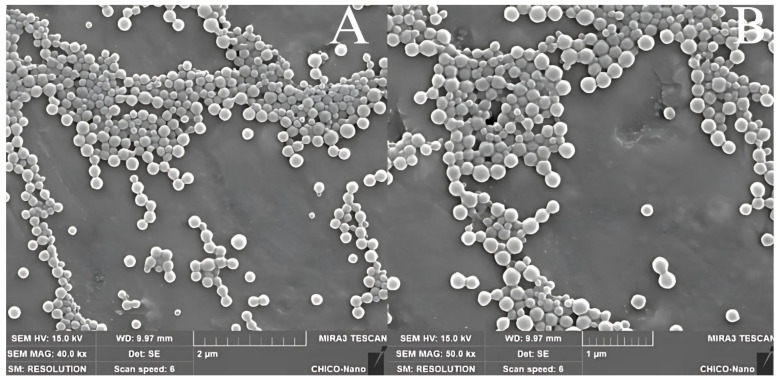
SEM images of QUER-ZNP-CH nanoparticles. (**A**) Morphology of nanoparticles at 40.0 k× magnification. (**B**) Morphology of nanoparticles at 50.0 k× magnification.

SEM analysis (Figure 4) confirmed that the QUER-ZNP-CH nanoparticles exhibit a predominantly spherical morphology with a relatively uniform size distribution and smooth surface.

#### 3.1.4. Long-Term Physicochemical Stability of Nanoparticles

The nanoparticles (QUER-ZNP-CH) were stored lyophilized at 4 °C for 12 months, and no significant variations were observed in average particle diameter, PDI, zeta potential, or drug content (Table 2).

#### 3.1.5. In Vitro Release Kinetics of QUER Encapsulated in Zein Nanoparticles

**Figure 5:**
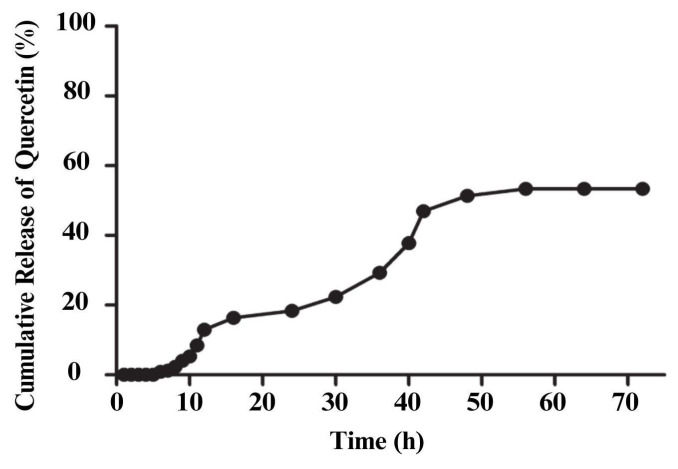
Release kinetics of QUER from zein/chitosan nanoparticles.

The in vitro release profile of QUER from QUER-ZNP-CH, evaluated by dialysis method in PBS buffer (pH 7.4) over 72 h, revealed sustained release over time. During the first 10 h, the cumulative release was below 20%, characterizing an initial slow phase. Subsequently, between 10 and 42 h, a continuous increase in release was observed, reaching approximately 55%. From 48 h onward, the release profile stabilized, remaining close to 60% until the end of the experiment, as illustrated in Figure 5.

### 3.2. In Vitro Antibacterial Activity

#### 3.2.1. Evaluation of Antibacterial Activity

The experiments demonstrated that both non-encapsulated QUER and QUER encapsulated in zein/chitosan nanospheres exhibit antibacterial properties (Table 3). The MIC of QUER ranged from 125 to >250 μg/mL, and the MBC was >250 μg/mL for all tested strains. The MIC of QUER-ZNP-CH ranged from 0.97 to 7.81 μg/mL, and the MBC ranged from 3.90 to 31.25 μg/mL. The MIC and MBC values for the encapsulated molecule showed a 32 to 64-fold reduction compared to the non-encapsulated form, indicating greater therapeutic efficacy with the use of this nanosystem.

#### 3.2.2. Evaluation of In Vivo Infection in *T. molitor* Model 

**Figure 6:**
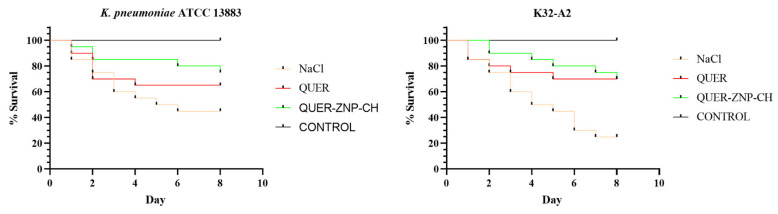
Evaluation of the survival rate of *Tenebrio molitor* infected with *Klebsiella pneumoniae* and treated with QUER and nanoencapsulated QUER. QUER: quercetin; QUER-ZNP-CH: zein nanoparticles coated with chitosan containing quercetin; CONTROL: Uninfected larvaes. ATCC: American Type Culture Collection; K: *Klebsiella pneumoniae* resistant to antimicrobials.

When analyzing the in vivo effect of *K. pneumoniae* infection and the therapeutic effect of QUER and QUER-ZNP-CH, a rapid progression of infection was observed in the infected group without treatment (NaCl), with a drop in survival rate on the first day. On the second day, survival was reduced to 60–75%, reaching only 45% by the end of the experiment. In contrast, the groups treated with the MIC of QUER and QUER-ZNP-CH showed survival rates higher than the control, remaining above 60% in the QUER-treated group and above 70% in the QUER-ZNP-CH-treated group, suggesting a relevant antibacterial and protective effect of QUER-ZNP-CH (Figure 6).

### 3.3. Antibiofilm Activity

**Figure 7:**
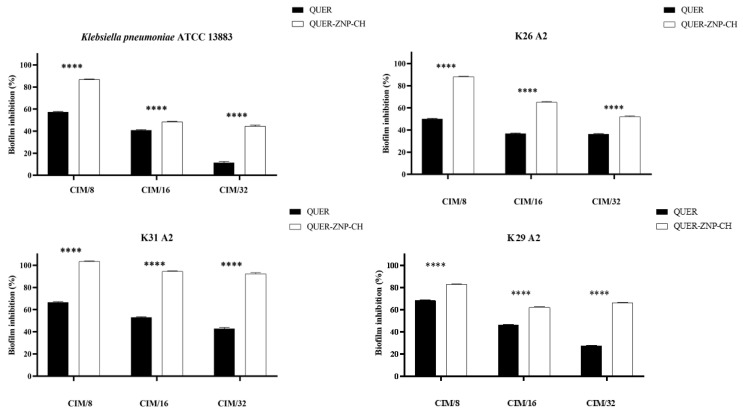
Evaluation of biofilm inhibition by QUER and QUER-ZNP-CH against *K. pneumoniae* isolates (%). Two-way ANOVA shows a highly significant difference between treatments (**** *p* < 0.0001). QUER: quercetin; QUER-ZNP-CH: zein nanoparticles coated with chitosan containing quercetin; MIC: Minimum Inhibitory Concentration; MBC: Minimum Bactericidal Concentration; ATCC: American Type Culture Collection; K: *Klebsiella pneumoniae* resistant to antimicrobials.

**Figure 8:**
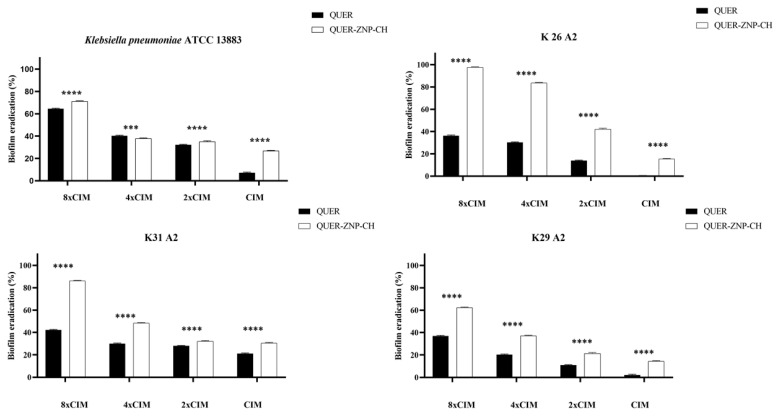
Evaluation of biofilm eradication by QUER and QUER-ZNP-CH against *K. pneumoniae* isolates (%). Two-way ANOVA shows a highly significant difference between treatments (*** *p* < 0.001, **** *p* < 0.0001). QUER: quercetin; QUER-ZNP-CH: zein nanoparticles coated with chitosan containing quercetin; MIC: Minimum Inhibitory Concentration; MBC: Minimum Bactericidal Concentration; ATCC: American Type Culture Collection; K: *Klebsiella pneumoniae* resistant to antimicrobials.

For these assays, only strains that produced moderate or strong biofilms were used, including *K. pneumoniae* ATCC 13883, K26-A2, K29-A2, and K31-A2. The biofilm inhibition potential of free QUER ranged from 40 to 60%, while QUER-ZNP-CH exhibited inhibition of 60–100% (Figure 7). Meanwhile, the biofilm eradication potential showed eradication values of 0–60% for free QUER, and 25–95% for QUER-ZNP-CH (Figure 8). These results demonstrate the enhancement of biofilm inhibition and eradication following QUER encapsulation.

### 3.4. Scanning Electron Microscopy of Biofilm

**Figure 9:**
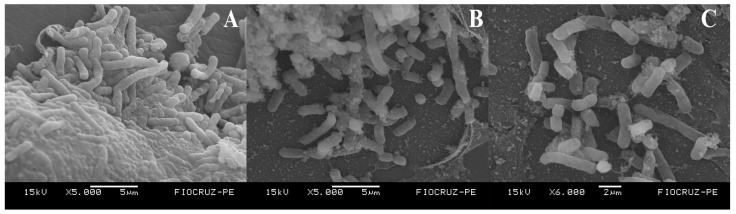
SEM of *K. pneumoniae* K26-A2. (**A**): Control without treatment; (**B**): Treatment with QUER; (**C**): Treatment with QUER-ZNP-CH.

Scanning Electron Microscopy (SEM) allowed the analysis of the efficacy of QUER-ZNP treatment in eradicating biofilm formation by *K. pneumoniae*, highlighting differences between the control group (Figure 9A) and treatments with non-encapsulated QUER (Figure 9B) and encapsulated QUER in zein/chitosan nanospheres (Figure 9C).

In Figure 9A, corresponding to the biofilm of the clinical isolate *K. pneumoniae* K26-A2 without treatment, a thick extracellular polymeric substance (EPS) matrix is observed, associated with a high density of bacterial cells, forming a highly organized biofilm structure. In Figure 9B, with the biofilm treated with QUER, the presence of EPS is noted, along with only a few disaggregated cells. On the other hand, in Figure 9C, after treatment with QUER-ZNP-CH, there is a substantial reduction in the amount of EPS, accompanied by cellular damage and reduced cell size. SEM corroborates the results obtained from the biofilm eradication activity studies, highlighting the effectiveness of QUER-ZNP-CH treatment in biofilm disruption.

### 3.5. In Vitro and In Vivo Toxicity

#### 3.5.1. Hemolytic Activity

**Figure 10:**
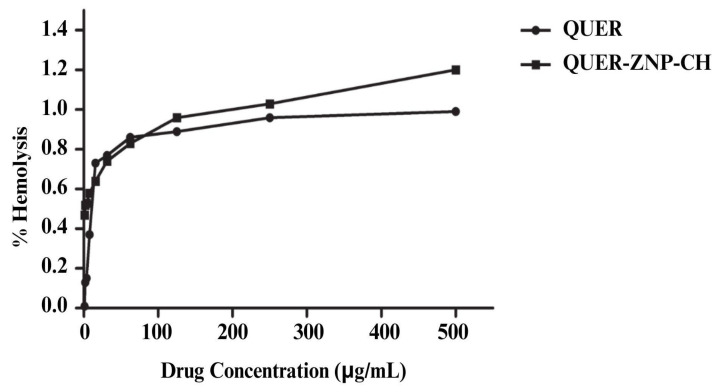
Evaluation of hemolytic activity of QUER and nanoencapsulated QUER on human erythrocytes. QUER: quercetin; QUER-ZNP-CH: zein nanoparticles coated with chitosan containing quercetin.

The hemolysis assay (Figure 10) revealed that both QUER and QUER-ZNP-CH exhibited hemolysis rates below 1.5% at all tested concentrations (10–500 µg/mL), characterizing them as non-hemolytic according to the American Society for Testing and Materials (ASTM) F756-17 criteria (<5%). It is noteworthy that ZNP-CH and PBS did not show toxicity (0%) at any concentration.

#### 3.5.2. Toxicity in *Tenebrio molitor* Model

**Figure 11:**
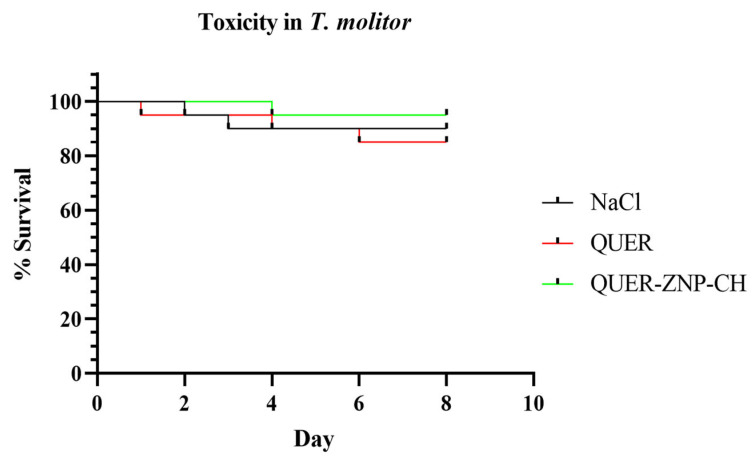
Evaluation of toxicity of QUER and nanoencapsulated QUER on *Tenebrio molitor* larvae. QUER: quercetin; QUER-ZNP-CH: zein nanoparticles coated with chitosan containing quercetin.

The in vivo toxicity assay performed on non-infected *T. molitor* larvae, subjected to the same concentrations used in the therapeutic tests, showed no significant behavioral changes and a high survival rate (Figure 11). The survival rate remained above 95% in the QUER-ZNP-CH group, which was higher than the QUER group (85%) and even the control group, where NaCl was administered (90%). These results indicate that, at the evaluated concentrations, the treatment with the nanostructure provided a protective effect and did not induce any obvious signs of acute toxicity in the studied model, reinforcing its biocompatibility and supporting its potential for future therapeutic applications, including in other experimental models.

## 4. Discussion

The %EE of ZNPs is directly influenced by the chemical properties of the bioactive molecule, the polymer concentration, and the preparation method employed. In the case of QUER, its hydrophobic nature favors spontaneous interactions with the apolar core formed by the self-association of hydrophobic amino acid residues in zein, promoting strong affinity with the polymeric matrix [33,34].

Polymer concentration also plays a crucial role in determining the structural density of the matrix, thereby impacting its ability to entrap the encapsulated molecule. When analyzed together, parameters such as hydrodynamic diameter, PDI, zeta potential, and encapsulation efficiency indicate the formation of a stable colloidal dispersion, with promising characteristics for oral and nasal administration routes [35,36].

Nanoparticles with sizes ranging from 100 to 500 nm exhibit an ideal profile for transcellular absorption and are particularly recognized by M cells in the intestinal epithelium and by cells of the respiratory tract, which facilitate their internalization through endocytosis-mediated mechanisms [37]. The narrow size distribution, evidenced by a PDI below 0.3, indicates high population homogeneity, an essential attribute for ensuring biological predictability and technical reproducibility. Furthermore, zeta potential values above ±30 mV reflect a sufficiently charged surface to promote electrostatic repulsion between particles, minimizing the risk of aggregation and ensuring physicochemical stability during storage and upon redispersion [38,39]

Moreover, the zeta potential showed only a slight decrease over time, from +49.2 mV on day 15 to +45.5 mV on day 365, remaining above +40 mV at all times—values indicative of strong electrostatic stability. The pH of the redispersion medium ranged between 5.1 and 5.5, with no evident impact on formulation stability. The quantification of QUER within the system revealed a slight decrease after six months, from 99.7% (day 15) to 98.9% (day 365). The maintenance of values close to 100% during the first 90 days reinforces the protective effect of the zein/chitosan polymer matrix on the encapsulated QUER. These results demonstrate that the nanoformulation maintains its physicochemical stability over a 12-month period [31,40].

The pH values of the formulations remained relatively stable over the 12-month storage period, ranging from 5.1 to 5.5, which reflects the chemical stability of the colloidal system. This slightly acidic pH is particularly important for maintaining the solubility and protonation of chitosan, which contributes to the preservation of its electrostatic interactions and mucoadhesive properties during storage and delivery. Additionally, from a physiological standpoint, mildly acidic pH conditions enhance the compatibility of the formulation with biological systems. In oral delivery, such pH helps maintain quercetin stability and improve mucosal absorption, especially in the upper gastrointestinal tract. In topical applications, a pH close to 5.5 is considered skin-friendly and helps reduce irritation while preserving the bioactivity of chitosan [40,41].

The FTIR spectra revealed clear evidence of intermolecular interactions within the QUER-ZNP-CH system. The broad O–H stretching band of quercetin (~3400 cm^−1^), attenuated and shifted in the formulation, strongly suggests the establishment of hydrogen-bonding networks between the hydroxyl groups of quercetin and both the amide carbonyl groups of zein and the protonated amino groups of chitosan [42,43]. This interaction is crucial, as it contributes to the stabilization of quercetin in a molecularly dispersed state within the nanoparticle matrix. The slight displacement of the Amide I band (~1650 cm^−1^) further indicates conformational adjustments in the secondary structure of zein, reflecting the accommodation of quercetin molecules in the protein backbone [44]. Additionally, the partial suppression of the aromatic C=C stretching vibrations (1600–1450 cm^−1^) confirms the participation of π–π stacking and hydrophobic interactions between the flavonoid rings and the nonpolar residues of zein, strengthening the supramolecular network [45].

These spectroscopic findings are corroborated by DSC and TGA. The absence of the sharp endothermic melting peak of crystalline quercetin (~170–180 °C) in the thermogram of QUER-ZNP-CH indicates that quercetin is no longer in its crystalline state, but instead exists in an amorphous or molecularly dispersed form [43,46]. This amorphization significantly enhances apparent solubility and reduces recrystallization propensity, properties highly desirable for hydrophobic compounds such as quercetin [47]. TGA curves demonstrated that the pure components degraded in multiple, distinct stages (lysine beginning at ~200 °C, zein at ~270 °C, and quercetin above 300 °C). In contrast, the formulation exhibited a unified degradation profile with a main mass-loss event concentrated at 300–330 °C, evidencing strong supramolecular cohesion within the nanoparticle system [48]. This altered thermal pathway supports the hypothesis that hydrogen bonding, van der Waals forces, and electrostatic interactions stabilize quercetin in the zein/chitosan matrix, conferring thermal robustness [44,47].

SEM micrographs provide morphological confirmation of these physicochemical interactions. The nanoparticles displayed predominantly spherical shapes with smooth, homogeneous surfaces, and crucially, no evidence of crystalline quercetin deposits such as lamellar or needle-like structures [43,49]. This observation reinforces the DSC data, confirming that quercetin is incorporated in an amorphous state. The uniformity observed under SEM correlates with the low polydispersity index (~0.25) obtained by DLS, validating the monodisperse nature of the colloidal system [46]. Moreover, the presence of a highly positive zeta potential (>+45 mV), conferred by the chitosan coating, supports the absence of aggregation by electrostatic repulsion, ensuring long-term colloidal stability [49,50].

The physicochemical stability of QUER-ZNP-CH was evaluated over a 12-month period under storage at 4 °C in lyophilized form. The system exhibited excellent colloidal and drug content stability, with no significant changes observed in the measured parameters. The mean particle diameter remained within the range of 343 to 352 nm throughout the entire period, with variations below 3%, indicating the absence of aggregation or significant particle growth. The PDI remained stable, ranging from 0.235 to 0.246, consistently below 0.3, reflecting the uniformity and monodispersity of the nanosystem [51,52,53,54,55].

The release profile observed for the QUER-ZNP-CH formulation demonstrates a sustained release system, consistent with the proposal of a controlled release system. The initial slow release (up to 10 h) suggests retention of QUER in the nanoparticulate matrix, which can be attributed to the hydrophobic nature of zein and the non-covalent interactions established with QUER, such as van der Waals forces, hydrogen bonding, and π-π interactions between the aromatic rings of the drug and the side chains of zein. The compact colloidal structure and the low initial aqueous solubility of zein contribute to this initial delay [32,56,57,58].

Chitosan also acts as an additional diffusion barrier, slowing down the influx of water and the efflux of encapsulated QUER. Moreover, its cationic nature can favor electrostatic interactions with anionic groups present on the surface of zein or in the structure of QUER itself (pKa ≈ 6.7), contributing to the stability of the formulation and controlling the release [32,59].

The gradual increase in release between 10 and 42 h is likely due to a rearrangement of the polymeric matrix in the aqueous medium, with progressive hydration and relaxation of the zein structure, facilitating the drug diffusion via a predominantly Fickian diffusion mechanism, typical of matrix polymer systems [14]. This behavior suggests that the system approaches a release kinetics pattern of the Higuchi or Korsmeyer-Peppas type, characteristic of systems with drug release by diffusion in a solid polymeric matrix [60].

The stabilization observed after 48 h, with a cumulative release close to 60%, indicates that the remaining fraction of the drug is strongly trapped or associated with less accessible internal regions of the matrix. This fraction may depend on slower processes, such as zein degradation or system solubilization under specific conditions. Such behavior is desirable in therapeutic strategies aimed at avoiding plasma concentration peaks, prolonging action time, and reducing side effects, especially for antioxidant compounds like QUER [52].

QUER is a flavonoid widely distributed in various fruits and vegetables, and has been extensively studied due to its antioxidant, antimicrobial, anti-inflammatory, antiviral, and anticancer properties [61,62]. Its antimicrobial activity is primarily attributed to mechanisms such as disruption of cell walls, destabilization of bacterial membranes, inhibition of nucleic acid synthesis, and reduction in intracellular enzymatic activity [63,64].

Given the antimicrobial potential of this molecule and the need for encapsulation, recent studies have shown that QUER-loaded nanoparticles exhibit broad-spectrum antimicrobial activity against both Gram-positive and Gram-negative bacteria, including *S. aureus*, *E. coli*, *Salmonella*, *Listeria*, and *Vibrio* spp. [65,66,67,68,69,70]. These studies demonstrate that nanostructured systems enhance the antibacterial effect of QUER through mechanisms such as controlled release, interaction with bacterial biofilms, and induction of structural membrane damage, confirmed by morphological analyses and a reduction in MIC values after encapsulation.

Encapsulation of therapeutic agents in polymeric nanoparticles, such as PLGA systems, has already demonstrated clinical promise in chronic diseases like pulmonary fibrosis, reinforcing the importance of controlled-release nanoplatforms [71]. Such controlled delivery strategies could similarly enhance the systemic bioavailability and therapeutic efficacy of QUER in the context of multidrug-resistant infections.

These results are consistent with the findings presented here, in which QUER encapsulation enhanced its antibacterial activity by 32 to 64-fold. Additionally, the encapsulation of QUER improves its physicochemical stability, protecting it from degradation caused by light, oxygen, and temperature, thus prolonging the shelf life of its biologically active form [72,73,74]. Furthermore, although the ZNP-CH nanosystem alone does not exhibit antimicrobial activity [15], the positive surface charge of the nanoparticles, resulting from chitosan coating and its intrinsic cationic nature, may potentiate the antimicrobial activity of QUER by destabilizing the negatively charged bacterial outer membrane. This facilitates the access of the bioactive molecule to its intracellular bacterial targets [17,75].

Therefore, the enhanced antibacterial activity of QUER-ZNP-CH may also be attributed to prolonged interaction with bacterial cells, enabling sustained action of QUER at its intracellular targets. This strategy could be a useful platform subject to further validation for the development of nanoparticulate antimicrobial systems aimed at treating bacterial infections caused by antimicrobial-resistant *K. pneumoniae,* [76,77,78].

The use of *T. molitor* as an alternative in vivo model has proven efficient for evaluating the therapeutic efficacy of antimicrobial compounds, as its larvae exhibit characteristics such as a body temperature range between 25 and 37 °C, similar to that of the human body, as well as structural and functional homologues in the immune system [79,80,81]. The increase in survival rates in the treated groups, particularly in the group treated with QUER-ZNP-CH, supports the results observed in vitro, given the antibacterial potential of this formulation. Thus, the incorporation of QUER into QUER-ZNP-CH may enhance the bioavailability and distribution of the active compound, reflecting in vivo antimicrobial effects, as evidenced by the higher survival rate of infected larvae treated with QUER-ZNP-CH. This activity suggests the inhibition of growth or elimination of the pathogenic microorganism in the *T. molitor* infection model, promoting more lasting therapeutic effects compared to non-encapsulated QUER. While *T. molitor* serves as a convenient and ethically favorable invertebrate model for preliminary evaluation of QUER-ZNP-CH, it is important to acknowledge that subsequent testing in appropriate mammalian models will be required.

*K. pneumoniae* biofilms are a virulence factor associated with infectious processes in the human respiratory, gastrointestinal, and urinary tracts, leading to the development of invasive infections, particularly in immunocompromised and immunosuppressed patients [82,83,84]. In biofilms, *K. pneumoniae* cells exhibit increased resistance to antibiotic treatment and immune response due to the mechanical and chemical barrier provided by the extracellular matrix. Therefore, inhibiting biofilm formation and eradicating biofilms represent a relevant therapeutic strategy, especially through innovative drug delivery strategies [65,85].

The antibiofilm activity of QUER has already been reported, especially due to its anti-Quorum Sensing (QS) potential. QS is the communication system through which signaling molecules promote bacterial adhesion, exopolysaccharide production, and biofilm maturation [86]. Furthermore, this flavonoid can bind to genes related to biofilm regulation in some bacteria, altering their transcription and significantly reducing their expression levels [87].

The encapsulation of QUER enhanced its antibiofilm activity, as seen in Figure 7 and Figure 8, reaching inhibition and eradication values of up to 100% The increased activity of QUER in this nanosystem can be attributed to factors such as the positive surface charge of QUER-ZNP-CH, which allows QUER-ZNP-CH to bind to the surface of bacterial biofilms, facilitating drug delivery, and the particle size of QUER-ZNP-CH (less than 500 nm), which enables the nanostructures to enter the water channels and pores present in biofilms, allowing the release of the active ingredient at the core of the structure [88,89].

Previous studies also provide evidence that quercetin or quercetin-based nanoparticles can act against *K. pneumoniae* biofilms. Hooda et al. (2020) demonstrated that quercetin-impregnated silver nanoparticles produced larger inhibition zones against *K. pneumoniae* (ATCC 790603) than free quercetin. Similarly, Shabana et al. (2021) reported that quercetin-loaded mesoporous silica nanoparticles enhanced host resistance against *K. pneumoniae* in a Nile-tilapia infection model. These findings strengthen the rationale for the potential use of quercetin-loaded zein nanoparticles against *K. pneumoniae* biofilms [90,91].

Additionally, the morphological analysis by SEM showed significant disorganization of the extracellular polymeric matrix and alterations in cell integrity, reinforcing that the observed effects are not limited to the functional level but also extend to the three-dimensional architecture of the biofilm, confirming the physical-structural efficacy of the QUER-ZNP-CH formulation.

In the hemolysis assay, the hemolysis values observed for all tested concentrations remained below 1.5% for both free QUER and the nanoencapsulated formulation. According to ASTM F756-17 standards [92] materials that induce less than 5% hemolysis are considered non-hemolytic and, therefore, compatible with systemic applications. Although slightly higher values were observed for the nanostructured formulation at the highest concentrations, this slight increase can be attributed to the presence of positive charges on the surface of chitosan-functionalized nanoparticles, which favor electrostatic interactions with the erythrocyte membrane. However, these interactions did not result in hemolysis, suggesting that encapsulation helps modulate the direct contact of QUER with the cell membranes, reducing its potential pro-oxidant effects. Therefore, these data reinforce the hemocompatibility of the formulation and support its potential use in systemic or transmucosal routes, with adequate biological safety [93,94,95].

The in vivo model using *T. molitor* has emerged as a valuable alternative tool for preclinical toxicity screening due to its high sensitivity to nanomaterials, operational simplicity, low cost, and ethical relevance. Recent studies have shown that this insect is capable of responding to toxic stimuli from various sources, such as metallic nanoparticles, polymers, nanoplastics, and drugs, through behavioral, biochemical, and histological changes, yielding results comparable to those obtained in vertebrate models [95,96,97].

In the present study, administration of the QUER-ZNP-CH formulation to uninfected *T. molitor* larvae did not induce any evident signs of acute toxicity. The survival rate exceeded 95%, which was even higher than that observed in the control group (0.9% NaCl) and in the group treated with free QUER (85%), supporting the biocompatibility of the nanoformulation. These results suggest that nanoencapsulation contributed to mitigating potential toxic effects of the active compound by modulating its release and reducing direct exposure to sensitive cellular structures.

The trend observed in this model is consistent with previous reports on zein- and chitosan-based delivery systems, which demonstrate good in vivo tolerability and are recommended for initial safety evaluations of bioactive nanosystems [98,99,100]. Altogether, the data reinforce that the QUER-ZNP-CH formulation combines therapeutic efficacy with a robust safety profile, positioning it as a promising candidate for systemic or transmucosal applications, with potential for further evaluation in higher-tier preclinical models, such as mammalians.

## 5. Conclusions

In this study, QUER-ZNP-CH were successfully developed using the nanoprecipitation method. The formulation exhibited suitable physicochemical characteristics, including particle sizes around 345 nm, low polydispersity (PDI < 0.25), high encapsulation efficiency (≈99.9%), and a strongly positive surface charge conferred by chitosan. Structural and thermal analyses confirmed the amorphization of QUER and strong intermolecular interactions between the drug and the biopolymers, contributing to long-term colloidal stability and controlled drug release over 72 h.

The nanoencapsulation enhanced the antibacterial and antibiofilm activities of QUER against multidrug-resistant *K. pneumoniae* clinical isolates. This nanotechnology-based formulation reduced MIC and MBC values by up to 64-fold compared to free QUER and achieved up to 100% inhibition and 95% eradication of mature biofilms. These functional effects were corroborated by scanning electron microscopy, which revealed architectural disintegration of the biofilm matrix and loss of bacterial cell integrity. Moreover, the system demonstrated hemocompatibility and potential biological safety, with >95% survival in the *Tenebrio molitor* larval model.

Thus, QUER-ZNP-CH represents a scalable nanotechnology-based approach for the treatment of infections caused by antibiotic-resistant and biofilm-producing *K. pneumoniae*. These findings warrant further investigation to better assess the translational applicability of this system.

## Figures and Tables

**Table 1:** Physicochemical Characterization of QUER-ZNP-CH.

Formulation	[QUER] (mg/mL)	Ø (nm)	PDI	ζ (mV)	%EE
QUER-ZNP-CH	0.5	345.0 ± 0.9	0.248	+51.1 ± 0.2	99.9 ± 0.6

QUER: quercetin; QUER-ZNP-CH: zein nanoparticles coated with chitosan containing quercetin.

**Table 2:** Long-term stability of zein nanoparticles containing QUER functionalized with chitosan. Zeta potential, pH, and drug content data are expressed as mean ± standard deviation from three independent replicates (*n* = 3). Particle size and PDI values represent single measurements obtained from cumulative analysis.

Days	Ø (nm)	PDI	ζ (mV)	pH	Drug Content %
15	343.1 ± 0.4	0.239	+49.2 ± 0.3	5.3	99.7 ± 0.4
30	349.7 ± 0.7	0.246	+47.1 ± 0.7	5.1	99.9 ± 0.7
60	346.3 ± 0.7	0.235	+48.8 ± 0.7	5.1	99.8 ± 0.3
90	345.2 ± 0.5	0.237	+45.9 ± 0.8	5.2	99.6 ± 0.9
120	344.9 ± 0.9	0.241	+46.6 ± 0.1	5.3	99.1 ± 0.9
180	349.0 ± 0.8	0.239	+44.9 ± 0.4	5.5	98.5 ± 0.7
270	351.7 ± 0.9	0.242	+45.1 ± 0.5	5.5	98.6 ± 0.6
365	352.1 ± 1.0	0.244	+45.5 ± 0.9	5.3	98.9 ± 0.1

Ø: particle size; PDI: polydispersity index; ζ: zeta potential.

**Table 3:** Antibacterial activity of QUER and QUER encapsulated in zein nanoparticles coated with chitosan.

	QUER		QUER-ZNP-CH	
	MIC	MBC	MIC	MBC
	μg/mL			
*K. pneumoniae* ATCC 13883	250	>250	3.90	15.6
*K. pneumoniae* ATCC 700603	250	>250	7.81	15.6
*K. pneumoniae* K25-A2	125	>250	1.95	7.81
*K. pneumoniae* K26-A2	>250	>250	7.81	31.25
*K. pneumoniae* K29-A2	>250	>250	7.81	31.25
*K. pneumoniae* K31-A2	>250	>250	3.90	7.81
*K. pneumoniae* K32-A2	125	>250	0.97	3.90

QUER: quercetin; QUER-ZNP-CH: zein nanoparticles coated with chitosan containing quercetin; MIC: Minimum Inhibitory Concentration; MBC: Minimum Bactericidal Concentration; ATCC: American Type Culture Collection; K: *Klebsiella pneumoniae* resistant to antimicrobials.

## Data Availability

The original contributions presented in this study are included in the article. Further inquiries can be directed to the corresponding authors.
